# Preoperative evaluation of renal artery in patients with renal tumor: Using noncontrast-enhanced magnetic resonance angiography

**DOI:** 10.1097/MD.0000000000005025

**Published:** 2016-10-21

**Authors:** Liangsong Zhu, Guangyu Wu, Jianfeng Wang, Jiwei Huang, Wen Kong, Yonghui Chen, Wei Xue, Yiran Huang, Jin Zhang

**Affiliations:** a Department of Urology; b Department of Radiology, Ren Ji Hospital, School of Medicine, Shanghai Jiaotong University, Shanghai, People's Republic of China.

**Keywords:** noncontrast-enhanced magnetic resonance angiography, partial nephrectomy, renal artery reconstruction, renal tumor

## Abstract

To investigate the feasibility of the noncontrast-enhanced magnetic resonance angiography (NCE-MRA) to evaluate renal arteries before partial nephrectomy (PN).

Retrospective analyzed 479 patients who underwent renal surgery between January 2013 and December 2015 with NCE-MRA or computed tomographic angiography (CTA) renal artery image reconstruction preoperative in our department. The renal artery reconstruction score (RARS) was based on the level of artery visualization in a 4-class criterion, and the R.E.N.A.L nephrometry score (R.E.N.A.L), arterial based complexity (ABC) were also analyzed.

Of the 479 patients, the overall-lever RARS was 3.62, and the average in 2 groups was no significant difference (NCE-MRA vs CTA, *P* = 0.072). The performance of NCE-MRA in PN group was similar with CTA. Further comparison demonstrated that the efficiency of NCE-MRA in moderate- or low-degree tumor according to the R.E.N.A.L and ABC complexity less than 3S was equal to CTA. However, high degree (*P* < 0.001), 3S (*P* = 0.027), or 3H (*P* < 0.001) would affect the imaging of renal artery. Intragroup analysis showed that tumor complexity such as max tumor size (*r* = −o.351, *P* < 0.001), R.E.N.A.L (*r* = −0.439, *P* < 0.001), and ABC (*r* = −0.619, *P* < 0.001) were closely correlated with the NCE-MRA performance. The images of 2 sides of the kidney were compared in single person as well, which was meaningful for NCE-MRA patients only (NCE-MRA, *P* < 0.001; CTA, *P* = 0.182).

The renal artery reconstruction performed by NCE-MRA is feasible and has a similar achievement in the PN potential recipients, with a lower side effect, and meets the requirements for making surgical decision. It has a broad application prospect in clinical practice; however, it still needs to further improve the ability in more complex tumors.

## 1 Introduction

The minimally invasive partial nephrectomy (PN) is routinely performed at many centers worldwide^[1]^ as a standard treatment for small renal tumors. Many systems have been applied to assess the complexity of the surgical approach, such as R.E.N.A.L nephrometry score (R.E.N.A.L) and arterial based complexity (ABC).^[2,3]^ Furthermore, noninvasive visualization of renal arteries is widely used by computed tomographic angiography (CTA) or magnetic resonance angiography (MRA), which is very important for preoperative evaluation of the anatomy of renal tumor and detecting the exceptional artery.

Previous study reported that 3-dimensional (3D) renal artery reconstruction images based on CTA could facilitate the management of intrarenal vasculature to the tumor,^[4]^ and that spiral CTA reconstruction was more accurate than MRA for renal arterial anatomy,^[5]^ so it becomes the golden standard for preoperative assessment in patients with renal tumor. However, CTA may induce a hypersensitivity reaction or nephrotoxicity because of the contrast agent,^[6,7]^ at the same time, radiation exposure inevitably occurred during the procedure. It is very necessary to develop a more secure method to alternate the conventional CTA. To our knowledge, another technology noncontrast-enhanced MRA (NCE-MRA) has been used to evaluate abdominal vessel. Some articles have showed that NCE-MRA was performed to be a valid method to diagnose the renal artery stenosis,^[8,9]^ but the study of NCE-MRA to assess the renal tumor's vascularity was limited. The purpose of this study was to investigate the equivalence of the NCE-MRA so as to estimate whether renal artery can achieve the similar surgical requirements compared with CTA.

## 2 Materials and methods

**Figure 1:**
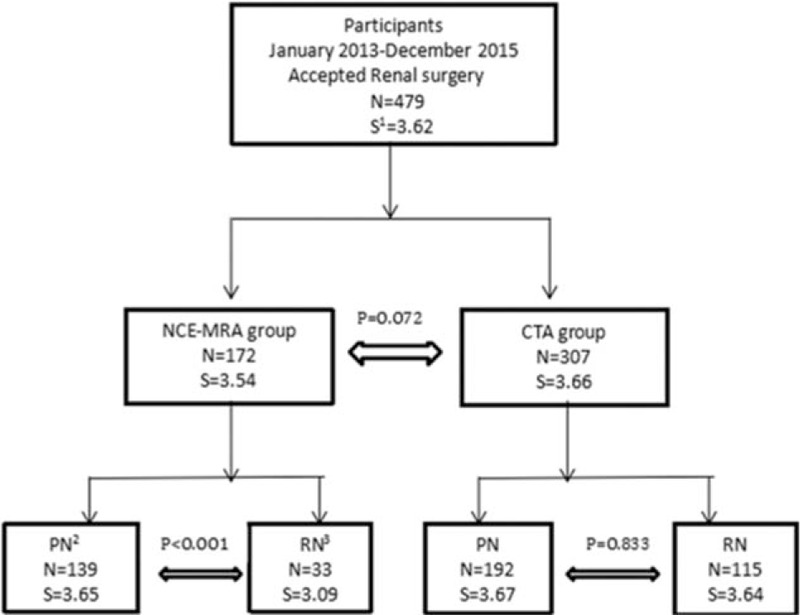
All 479 patients selected and divided into 2 groups, each group counted number and average score, respectively. Different surgical approaches were separated, PN and RN also analyzed as main group. S = renal reconstruction score, PN = partial nephrectomy, and RN = radical nephrectomy.

The study was approved by our institution ethics committee. Between January 2013 and December 2015, 479 patients (331 PN and 148 radical nephrectomy [RN]) were included in this study (Fig. 1) and all participants met the following inclusion criteria: the patient had complete information, bilateral renal carcinoma patients had been excluded, and no past surgery history. A total of 172 of them accepted NCE-MRA, 110 men and 62 women; median age and range, 55 (22–89) years, the rest of 307 patients adopted CTA, 196 men and 111 women; median age and range, 56 (21–84) years.

### 2.1 CT and MRI protocol

All computer tomographic (CT) examinations were performed by using a 64-MDCT scanner (VCT LightSpeed, GE Healthcare, USA). Images in 4 phases were obtained in a craniocaudals direction. The scanning included the diaphragm to the lower pole of the kidneys. Contrast-enhanced images were obtained after intravenous administration of 150 mL of nonionic contrast medium (Iopamiro, Bracco, Milan, Italy). The scanning parameters of each phase were 110 to 380 mA of tube using current modulation software, 1.25-mm collimation, and a pitch of 1.375. Unenhanced nephrographic phase and excretory phase scans were reconstructed as 1.25-mm sections. The arterial phase images were reconstructed at 0.725-mm intervals. The renal artery was evaluated based on arterial phase.

NCE-MRA was performed on a 3.0-T magnetic resonance scanner (Ingenia, Philips, Best, The Netherlands), the noncontrast MRA sequence is a 3D multishot balanced fast field echo sequence with a brenth-trigger. Data acquisition is accelerated by using a parallel imaging sensitivity encoding factor of 1.2. The following parameters were used: repetition time/echo time = 5.9/2.7 ms, 27° flip angle, voxel size 1.2 × 1.2 × 1 mm^3^, turbo field echo factor of 44, 2 signal averages and a bandwidth of 769-Hz pixel delay time of 325 ms, spectral presaturation with inversion-recovery fat suppression, and a regional saturation technique slab to suppress signal from the inferior vena cava, renal vein, and intestines.

### 2.2 Image analysis

All images were evaluated by 2 independent radiologists (with 3 and 4 years of experience in radiology, respectively). CTA and NCE-MRA were presented to each reader in a random fashion. All readouts were performed at an imaging workstation. The individual use of windowing, multiplanar reformations, maximum intensity projection reformats, and volume rendering were allowed. The inter-readers agreement was achieved in every case.

The performance of image was based on the visualization of renal arteries and was divided into 4-classes criterion. The standard were as follows: 1 point, main artery was detected; 2 points, branch artery was determined; 3 points, segment artery could be measured; and 4 points, interlobar artery was clearly visualized. All of the images renal artery reconstruction score (RARS) and R.E.N.A.L, ABC grade were independently reviewed by 2 readers, 1 urologist, and 1 radiologist. Readers were blinded to patients’ messages, surgical approach, clinical outcomes, and pathological feature.

### 2.3 Statistical analysis

The performance of NCE-MRA and CTA in subgroups were compared by chi-square test. The patients and tumor characteristics among the 2 groups were evaluated by using Kruskal–Wallis analysis of variance tests for rank variables as well as the independent-samples *t* test for continuous variables. The factors would be associated with the performance of NCE-MRA assessed by bivariate spearman correlation analysis. Mann–Whitney test was used to compare bilateral images’ RARS. All reported *P* values are 2-sided, of which *P* value <0.05 was considered statistically significant. All statistical analysis was performed by IBM SPSS Statistic 19.0, Chicago, USA.

## 3 Results

Overall condition is illustrated in Fig. 1. The mean RARS in the study was 3.62, and there was no significant difference between NCE-MRA and CTA in the average level (*P* = 0.072). Baseline characteristics, Table 1, showed the patient characteristic of the entire cohort. All 479 patients included 306 (63.9%) men and 173 (36.1%) women, with a median age 56 years (range 21–89 years). The body mass index of the total group was 24.9 ± 3.08. The max tumor size was 4.51 ± 2.38 cm. There was no significant difference between 2 groups in terms of patient characteristics and max tumor diameter. Patients were restratified into 3 groups (Low, Moderate, and High) according to R.E.N.A.L nephrometry, and 4 groups (1, 2, 3S, and 3H) according to ABC complexity. Also, the result of RARS was scored in 3 groups (2, 3, and 4) according to this study's 4-classes criterion as mentioned before, and there was no patient at 1 point at all. There was significant difference in the classification of R.E.N.A.L (*P* = 0.027), ABC (*P* < 0.001), as well as RARS (*P* = 0.024) in 2 groups. Table 2 indicated that 2 methods had no distinction in RARS when tumor was in the moderate-to-low R.E.N.A.L degree and complexity less than 3S in the light of the ABC. The efficiency of CTA was superior to NCE-MRA when the tumor became high degree (*P* < 0.001) or more complex (3S, *P* = 0.027; 3H, *P* < 0.001). Table 3 intragroup analysis demonstrated that the tumor's characteristics, such as R.E.N.A.L (*r* = −0.439, *P* < 0.001) and ABC (*r* = −0.619, *P* < 0.001) could clearly decrease the performance in NCE-MRA group, and max tumor size (*r* = −0.351, *P* < 0.001) explained that bigger the tumor grew, the more difficult the renal arteries visualized. CTA group was influenced as well. We compared the bilateral renal arteries of every patient (Table 4), the affected side was scored less than the uninjured in NCE-MRA group (*P* < 0.001); however, there was no significant difference in CTA group (*P* = 0.184). The uninjured kidney of NCE-MRA group was scored similar with CTA indeed.

**Table 1:**
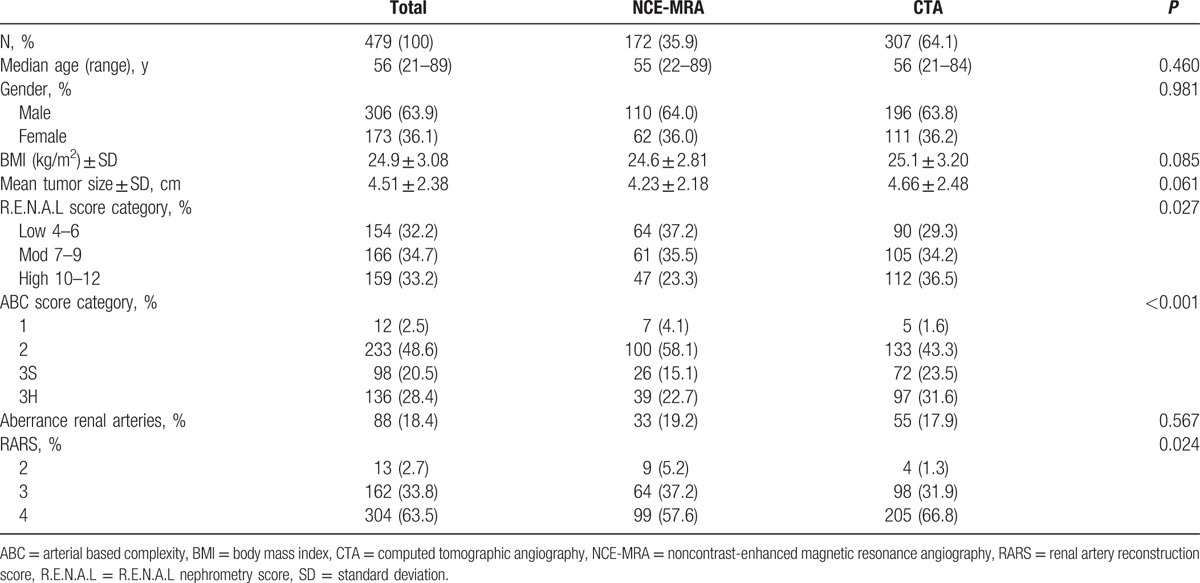
Patient, tumor characteristics, and renal reconstruction score according to the images.

**Table 2:**
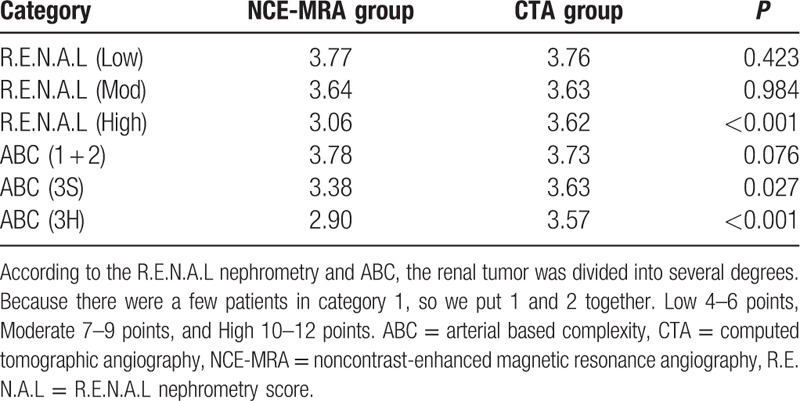
RARS in different tumor characteristic.

**Table 3:**
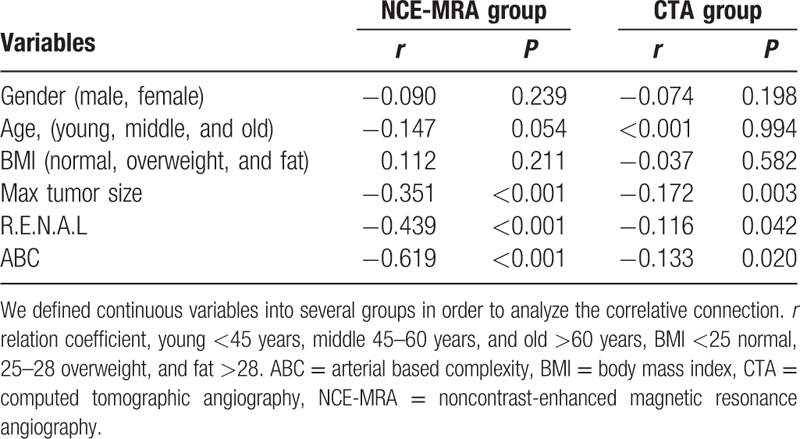
Factors associated with final reconstruction score of NCE-MRA.

**Table 4:**
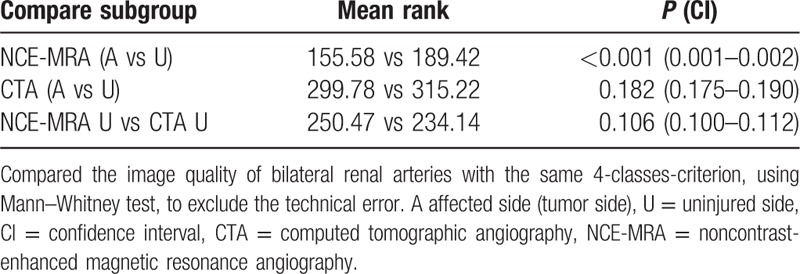
The bilateral RARS of each group were analyzed.

## 4 Discussion

Because of the prevalent use of abdominal imaging during routine health examination, the renal tumor incidence has been increasing over past several decades,^[10]^ laparoscopic or robotic assisted partial nephrectomy is now widely used as a standard option for these cases. A trifecta outcomes were revealed during PN, consisting of negative tumor margins, maximum renal functional preservation, and no urological complication.^[11]^ In order to achieve the trifecta goal, we need to have a good understanding of renal anatomy, especially the tumor's location and feeding artery.

CTA and contrast-enhanced MRA (CE-MRA) play an important role in preoperative assessment and decide which artery to clamp, Shao et al^[12]^ have shared their experience with segmental renal artery clamping during laparoscopic partial nephrectomy, or take anatomic targeted dissection and superselective control of tumor-specific renal artery,^[13]^ which Ng et al have tested before. However, the contrast agents of CTA have some renal damage,^[14]^ and Gadodiamide agents of CE-MRA may also be associated with nephrogenic systemic fibrosis.^[15]^ This is very important and valuable to investigate the feasibility of NCE-MRA to determine the renal tumor's vasculature for surgical decision-making.

The efficiency of NCE-MRA in our study was satisfactory. The average score was 3.56, which means that we can visualize the third level of renal artery called the segment artery, and there was no significant difference compared with CTA protocol. In the initial stage, during the PN, the main artery was routinely clamped to minimize blood loss and to create a relatively bloodless condition for tumor excision and renal reparation, which just needs to find out the location of the trunk artery at all. Table 1 shows that all participants scored more than 1 point, so this can be easily achieved by both CTA and NCE-MRA in our study. However, main artery clamping leads to ischemic damage of the rest renal parenchyma, and increasingly prolonged ischemia times may be more likely to cause acute kidney dysfunction.^[16,17]^ In order to manage the ischemia insult, the selected clamping of the pertinent segmental artery was used to reduce the damage.^[18]^ We need to determine the secondary artery at least, and we can definitely mark out the branch artery by NCE-MRA, as well as 94.7% of patients will be more subdivided into the third level. Therefore, it will be relatively simple to make decision by means of NCE-MRA protocol. When it comes to terms of moderate-to-low degree of renal tumor according to the R.E.N.A.L, and scored complexity less than 3S in accordance with the ABC, the effectiveness of NCE-MRA is similar to CTA.

**Figure 2:**
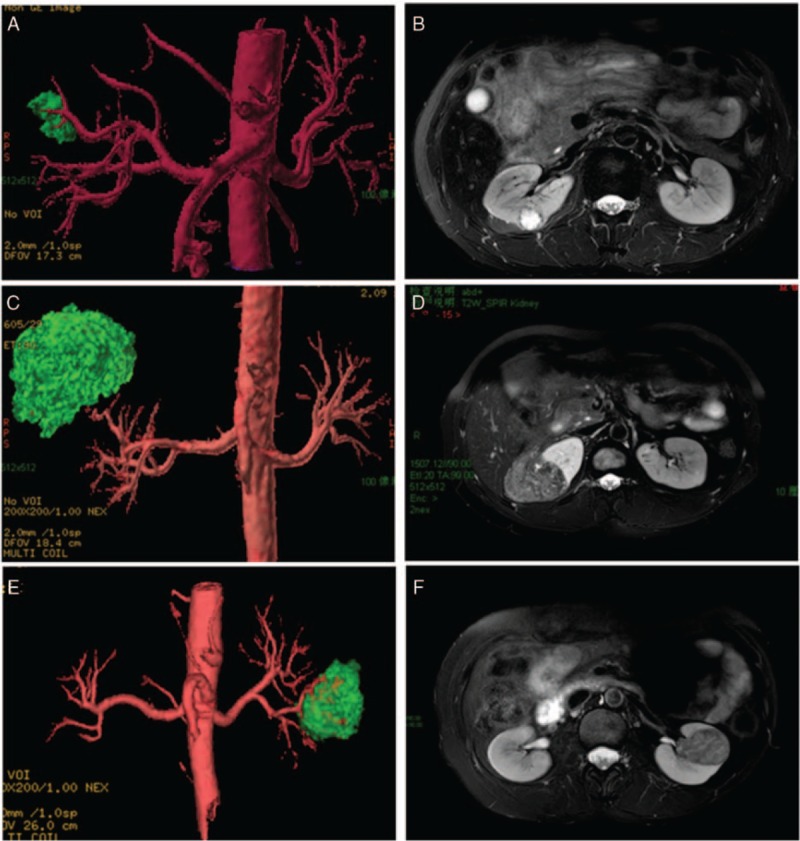
Showed that (A) and (B) (low), (C) and (D) (moderate), (E) and (F) (high) represented 3 degrees of renal cell carcinoma according to R.E.N.A.L nephrometry score, respectively, and each patient got 4-class renal artery visualization.

Nevertheless, we have observed (Table 2) a strong trend in our study that the more complex the tumor grows, the less completeness renal artery reconstructed. If the tumor has intricate anatomic features (max tumor size >4, endophytic, high nephrometry score, and category 3S or 3H in ABC system which means near the renal hilus), the RARS will decline in NCE-MRA technology. Intragroup comparsion was properly carried out and the result in Table 3 illustrated that the tumor size, R.E.N.A.L, and ABC will noticeably decrease the level of vascular imaging. Because, when performing the NCE-MRA, after background tissue suppressing, tumor's location and the relationship with the blood vessels could impede the inflow of unsaturated arterial blood, so that high vascular signal will be weaker in the subordinate artery during the same delay time. The CTA group is affected as well. Attributed to contrast-medium offering a good tracing ability in the arterial phase, CTA group has no obvious negative correlation, and hence, CTA has its undoubted advantages. On the other hand, the patients with complicated tumor are more likely to accept RN surgery. In addition, if the tumor is small and exophytic, the fourth lever or even arcuate artery can be showed. Thus, NCE-MRA still meets the requirement of superselective clamping skill (Fig. 2).

It is worth mentioning that, compared to CT, magnetic resonance imaging (MRI) may be better in detecting perirenal fat invasion and evaluating the venous thrombus as well as distinguishing the benign thrombus from tumor thrombus.^[19]^ Additional MRI scan during the NCE-MRA procedure can get 2 results at 1 shot.

**Figure 3:**
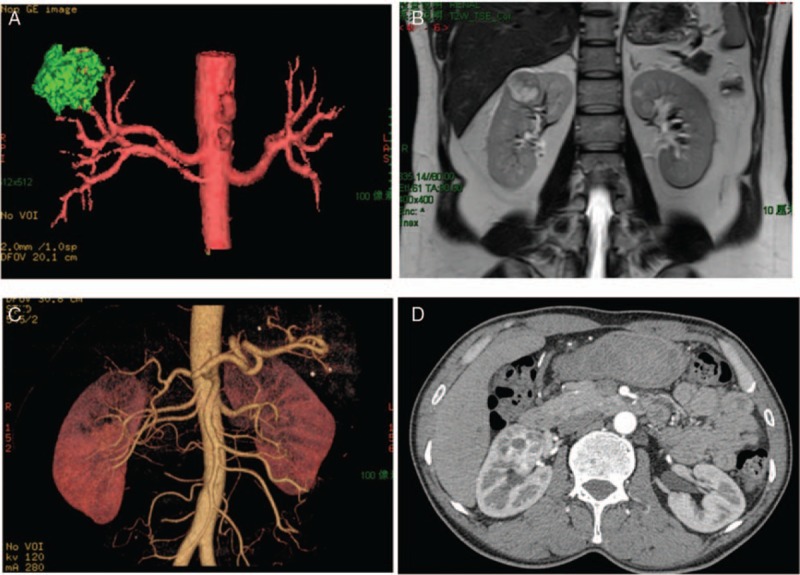
Showed the similar ability of detecting aberrance renal arteries in noncontrast-enhanced magnetic resonance angiography (A) and (B) and computed tomographic angiography (C) and (D).

Previous study has compared the usefulness of NCE-MRA with CTA in preoperative evaluation of potential living renal donors and stated that an optimized NCE-MRA could be substituted by CTA for preoperative evaluation of the vessel anatomy of multiple arteries.^[20]^ In our study, the rates of detecting the aberrance renal arteries in 2 groups were the same (Fig. 3). More important, NCE-MRA does not require ionizing radiation or the use of contrast agent to avoid the risk of unpredictable secondary affect^[21,22]^; therefore, borderline renal function patients, bilateral renal tumors or single kidney patients, pregnant patients, and patients who after PN need to accept the follow-up examinations are the candidates of NCE-MRA.

Our study had several limitations. First of all, this was a retrospective study and the work was not performed on 1 person who accepted both NCE-MRA and CTA, which may cause bias. A few patients accepted 2 technologies preoperatively at that time, which may be considered the excessive medical treatment. However, the number of patients in our work was relatively large, and we divided the patients into several subgroups in order to reduce the difference by means of comparing similar tumor characteristic respectively. Theoretically, the 4 levels or more arteries objectively exist in every patient, and our purpose was to find out whether NCE-MRA could indicate the tumor's artery for surgical assessment and which lever of renal artery could determine as CTA at all. The performance about completeness of renal artery reconstruction and morphological differences in 2 methods were not that concerned, and the result was believable and the feeding artery was confirmed during the operation as well as aberrance artery.

What is more, this work was a single-center study. We investigated the ability of NCE-MRA in PN preoperative assessment only, but the performance in benign and malignant tumors was not clearly defined. Thus, further prospective studies with a large population are necessary and need to be verified by multiple medical centers.

## 5 Conclusion

In conclusion, the NCE-MRA has undeniable advantage in renal artery visualization on account of the lower side effect and repeatability in a short term. Although, there are several factors associated with the performance of artery reconstruction, NCE-MRA is feasible and has a good achievement in preoperative risk assessment among the PN potential recipients. With the further development of technology, NCE-MRA may be considered as an alternative method for conventional CTA to evaluate renal artery in different complexity of renal tumor.
